# Exogenous spermidine enhances the photosynthetic and antioxidant capacity of citrus seedlings under high temperature

**DOI:** 10.1080/15592324.2022.2086372

**Published:** 2022-06-15

**Authors:** Xu Chao, Tang Yuqing, Liu Xincheng, Yang Huidong, Wang Yuting, Hu Zhongdong, Hu Xinlong, Liu Buchun, Su Jing

**Affiliations:** a Key Laboratory of Horticultural Plant Genetics and Physiology, Institute of Horticulture, Jiangxi Academy of Agricultural Sciences, Nanchang, P. R. China; b Institute of Environment and Sustainable Development in Agriculture, CAAS/National Engineering Laboratory of Efficient Crop Water Use and Disaster Reduction/Key Laboratory of Agricultural Environment, Ministry of Agriculture and Rural Affairs, Beijing, P. R. China; c Nanjing Institute of Environmental Sciences, MEE, Nanjing, P. R. China

**Keywords:** Antioxidants, chlorophyll fluorescence, gas exchange, heat stress, photosynthesis

## Abstract

Studies have not fully explained the underlying mechanism of spermidine-mediated heat tolerance. This study investigated the possible role of spermidine (Spd) in regulating citrus heat tolerance. The results showed that exogenous Spd effectively alleviated the limitation of high temperature (HT) on photosynthesis. Exogenous Spd increased the chlorophyll content, net photosynthetic rate, intercellular carbon dioxide concentration, stomatal conductance, maximum and effective quantum yield of PSII photochemistry, nonphotochemical quenching coefficient, and electron transport rate in citrus seedlings under HT stress, but declined the stomatal limitation value. In addition, Spd treatment promoted the dynamic balance of the citrus enzymatic and non-enzymatic antioxidants system. Spd application significantly increased the activity of superoxide dismutase, peroxidase, catalase, ascorbic acid, and glutathione and the expression level of corresponding genes at high temperature, while reducing the content of H_2_O_2_ and malondialdehyde. Therefore, our findings suggested exogenous Spd significantly ameliorated citrus physiological and photosynthetic adaptation under HT stress, thereby providing helpful guidance for citrus cultivation in HT events.

## Introduction

Because of global warming, frequent high temperature (HT) has become one of the major environmental threats that seriously limits plant growth, metabolism, and productivity.^1^ According to the latest IPCC synthesis report (IPCC 2021), the global surface temperature is supposed to increase by 1.5°C until the middle of the century and that would result in crop yield reductions of 2.5–16%. The most vital physiological and biochemical processes in plants are extremely susceptible to HT stress.^2^ The HT stress substantially decreases the photosynthesis capability and photosynthetic pigments content in plant leaves by Zhang *et al*.,^3^ reducing the photosynthetic activity in plants. The stress is associated with stomatal limitations, such as stomatal closure, and/or non-stomatal limitations. In the photosynthetic apparatus, these limitations include chlorophyll degradation, chloroplast ultrastructure damage, degradation of membrane, and the enzymatic proteins.^4^ The HT stress also causes oxidative damage in plant cells due to the excess production of reactive oxygen species (ROS), such as hydrogen peroxide (H_2_O_2_), hydroxyl radical (OH^·^), superoxide (O_2_^−^), among others etc.^5^ These damages result in cell membrane lipid peroxidation, discoloration of pigments, proteins inactivation, RNA and DNA molecules damage, which will eventually lead to plant death.^6^ Plants have natural defense mechanisms to resist oxidative stress to some extent by regulating underlying enzymatic and non-enzymatic antioxidant defense systems. Enzymatic antioxidants include superoxide dismutase (SOD), peroxidase (POD) and ascorbate peroxidase (APX), etc., which can maintain the balance between the production and scavenging of ROS by removing excess ROS in plants and protect plants from ROS-induced damage.^7,8^ Therefore, the photosynthetic capacity and antioxidant capacity are highly important for normal growth and development of plants under HT stress. Non-enzymatic antioxidants include ascorbic acid (AsA), glutathione (GSH), phenolic compounds, and tocopherols, which can regulate the functions of main cells and participate in the

removal of ROS produced by plants during stress.^9,10^

Polyamines (PAs) are the ubiquitous low-molecular-weight aliphatic amines that are involved in the regulation of plant growth and development.^11^ In higher plants, the major PAs are the diamine putrescine (Put), spermidine (Spd), and the two tetraamines, spermine (Spm) and thermospermine (T-Spm).^12^ Research in recent years showed that PAs are also positive regulators implicated in defense responses to various types of environmental stresses such as salinity, chilling, drought, heat stress, and heavy metals.^13,14^ The PAs also have the roles in stimulating seed germination, modulating root morphology, promoting flower bud differentiation, inhibiting shoot branching, and increasing fruit set rate.^15,16^ In large, plants experiencing abiotic stress tend to increase PA levels, which provides tolerance by changing hormone balance, inducing antioxidant enzymes, and stabilizing membrane integrity and function.^17^ Spd, a common PA in plants, is involved in alleviating the detrimental effects on plants of various abiotic stresses. The accumulation of Spd in plants under heat stress is results from the improvement of high-temperature resistance. The Spd-mediated protection has a close association with the maintained chloroplast and mitochondria ultra-structures. It enhances photosynthesis and HT tolerance in wheat.^18^ Moreover, the Spd pretreatment alleviates the negative effects of HT on the photochemical efficiency of the photosystem II (PSII) of lettuce seedlings.^19,20^ Furthermore, exogenous Spd regulates antioxidant enzyme activity, reducing superoxide anion rate and malondialdehyde (MDA) content to maintain plant cell membrane stability.^21^ Even so, the information regarding the effects of exogenous Spd on photosynthesis and the antioxidant machinery of heat-stressed plants is limited. Therefore, it is essential to obtain a better understanding of how Spd regulates the photosynthetic performance of plants under HT stress conditions.

Citrus is the most extensively produced tree fruit crop in southern China.^22^ The suitable temperature for citrus growth is 15–28°C, and thus the continuous high temperature in summer inhibits citrus growth and development.^23^ Nonetheless, to the best of our knowledge, few studies have been focused on the potential of Spd in improving the heat tolerance in citrus. This study explored the possible role of Spd in improving heat tolerance in citrus, based on changes in photosynthetic characteristics, gas exchange traits, chlorophyll fluorescence parameters, and stability of the antioxidant system.

## Materials and methods

### Plant material and growth condition

The conducted experiments were from July to September 2021 in the Venlo-type glasshouse of the institute of Horticulture in Jiangxi Academy of Agricultural Sciences, Nanchang, China. The experimental ground lies between latitude 28°51”N, longitude 115°95”E, and altitude of 53 m above sea level. Individual healthy and uniform three-year-old citrus seedlings (*Newhall navel orange* (Citrus × sinensis cv. Osbeck)), a lowbush citrus cultivar, which is popular in southern China, were planted in pots with a top diameter of 40 cm, a bottom diameter of 50 cm, and a height of 70 cm. The pots filled with sandy loam containing organic matter of 17.75 g kg^–1^, total nitrogen of 0.97 g kg^–1^, total phosphorus 1.82 g kg^–1^, total potassium 10.33 g kg^–1^, readily available phosphorus 17.92 mg kg^–1^, readily available potassium 136.47 mg kg^–1^, and pH 6.63. These all elements were the most suitable soil physical and chemical properties for citrus seedlings growth. Before the experiment, all plants were raised for 7d in an artificial climate chamber (A1000, Conviron, Canada) with day/night temperature of 28/18°C(day/night), light intensity of 800 μmol m^–2^ s^–1^, photoperiod of 12/12 h (light/dark), and relative humidity of approximately 65%.

After pre-treatment, put all pots in a completely randomized design in a factorial arrangement and seedlings then divided into the following four groups. Two groups were subjected to a temperature of 28/18°C (day/night) and sprayed with distilled water and 0.5 mmol L^–1^ Spd (0.5 mmol L^–1^ Spd is the empirical value based on the previous experiments of the research group), respectively [28/18°C + H_2_O (CK, normal control) and 28/18°C + 0.5 mmol L^–1^ Spd (Spd)]. The other two groups were subjected to HT treatment at 38/28°C and sprayed with distilled water and 0.5 mmol L^–1^ Spd, respectively [38/28°C + H_2_O (HT) and 38/28°C + 0.5 mmol L^–1^ Spd (HT + Spd)]. They uniformly spray-leaves on both sides until the solution on the leaves formed fine mist-like droplets. Each treatment consisted of five (5) pots and three replications of each experimental unit, with 60 pots in total. The environmental conditions of each artificial climate chamber were as follows: photoperiod of 12/12 (day/night), the illumination intensity of 800 μmol m^–2^ s^–1,^ and relative humidity of approximately 65%. Citrus seedlings were foliar sprayed with Spd purchased from Sigma-Aldrich every two days and this treatment continued for six days before the HT treatments in which Spd sprayed evenly on the leaf surface and the back of the leaf until the front, the back leaf surfaces were wet, and no liquid dripped. The collection of leaf samples was on the 3rd day of HT treatment and the relevant physiological indicators were determined.

### Measurement of chlorophyll content

Chlorophyll a (Chl*a*), chlorophyll b (Chl*b*), total chlorophyll, and carotenoid (Car) contents were determined according to the method reported by Arnon^24^ with minor modifications. A leaf sample of 0.2 g was ground in a mortar and placed into a 25 ml centrifuge tube containing 20 ml 95% acetone. The tube was sealed and placed in a dark place until the pigment in the leaves was completely extracted. Chl*a*, Chl*b*, and total Chl concentrations were measured using ultraviolet/visible absorption spectrophotometry (UV-9100, LabTech, China) and absorbance was measured at 663 nm, 645 nm, and 470 nm. The following formulae were used for calculations: Chl*a*= 12.7A_663_ – 2.59 A_645_, Chl*b*= 22.9A_645_ – 4.67A_663_, total Chl = 34.5× A_663×_1000, Car = (1000A_470 –_ 3.27 Chl*a*_–_ 104 Chl*b*) /229. The content of Chl and Car are expressed as mg g^–1^ (FM).

### Measurement of gas exchange parameters and light response curves

Gas-exchange parameters were measured by using the portable photosynthesis system (Li-6400XT, Li-COR Inc., USA), as described previously by Yang *et al*.^25^ Measurements were conducted between 9:00 and 11:00 hours. Before the measurement, each leaf was induced for 10 minutes in the leaf chamber (2 × 3 cm) with constant light intensity (800 μmol m^–2^ s^–1^). During the measurement, the light irradiation, leaf chamber temperature, and CO_2_ concentration were maintained at 800 μmol m^–2^ s^–1^, 25°C, and 400 μmol mol,^‒^^1^ respectively. The LI-6400 program recorded the Intercellular carbon dioxide concentration (*C*_i_), stomatal conductance (*g*_s_), and net photosynthetic rate (*P*_N_). The stomatal limit value (Ls) was calculated by the equation Ls = 1 − *C*_i_ /*C*_a_, where C_a_ represents the atmospheric CO_2_ concentration.^8^

The light response curves under different treatments were measured based on Hermann *et al*.^26^ The light intensity gradient of PAR was set as 0, 20, 50, 80, 100, 300, 500, 700, 900, 1100, 1200, and 1500 μmol m^–2^ s^–1^ for 120 s each. The environmental parameters in the leaf chamber were the same as when measuring the gas exchange parameters.

it was necessary to fit the light response curve to accurately obtain the parameters on the light response curve such as the light compensation point (LCP), the light saturation point (LSP), maximum net photosynthetic rate (*P*_Nmax_), and the apparent quantum yield (AQE), Light response curves were simulated by a non-orthogonal hyperbolic model (Equation 1) using SPSS 17.0 (SPSS Inc., Chicago, IL, USA) as described previously by Farquhar *et al*.^27^

(1) $$P_{N}(I)=\frac{\alpha I+P_{\max}-{\left[{\left(\alpha I+P_{\max}\right)}^{2}-4\theta \alpha IP_{\max}\right]}^{0.5}}{2\theta }-R_{d}$$

where, *P*_N_(*I*) is the net photosynthetic rate, *I* is the light intensity, θ is the curvature of the curve, and α is the slope of the plant photosynthesis versus light response curve at I = 0, also called initial quantum efficiency, *P*_max_ is the maximum net photosynthetic rate and R_d_ is the dark respiration rate.

### Measurement of chlorophyll fluorescence parameters

Chl fluorescence was measured using a pulse amplitude modulation fluorometer (PAM-2500, Walz, Germany) after leaves were dark-adapted for 20 min. The maximum quantum yield of PSII photochemistry (F_v_/F_m_), nonphotochemical quenching (NPQ), the effective quantum yield of PSII photochemistry (ΦPSII), and electron transport rate (ETR) were measured. Chl fluorescence parameters were calculated following the method reported by Jan *et al*.^28^

### Measurement of malondialdehyde (MDA) and hydrogen peroxide (H_2_O_2_)

MDA content was measured by the thiobarbituric acid (TBA) method described in a study conducted by Hodges *et al*.^29^ Firstly, 0.5 g of fresh leaves were ground with 5 ml of 10% trichloroacetic acid (TCA) in a mortar, and the extract was centrifuged for 20 min at 4,000 × g at 4°C. Next, 2 mL of the supernatant taken in a 5-mL centrifuge tube and 2 mL of 0.67% thiobarbituric acid (TBA) was added. The tube containing a well-mixed sample immersed in a boiling-water bath for 30 min, and re-centrifuged after cooling. The absorbance of the supernatant was recorded at 532, 450, and 600 nm using ultraviolet/visible absorption spectrophotometry (UV-9100, LabTech, China). The MDA content of expressed as μmol g^–1^.

H_2_O_2_ was determined by using the method reported by Wang *et al*.^30^ with minor modification. 0.5 g of fresh leaves homogenized with 5 mL of 0.1% (w/v) TCA in an ice bath. After centrifugation at 12,000 × g for 20 min, 0.5 mL of the supernatant was then added to 0.5 mL of 10 mM potassium phosphate buffer (pH 7.0) and 1 mL of 1 M potassium iodide. The ultraviolet/visible absorption spectrophotometry (UV-9100, LabTech, China) used again to measure absorbance of the supernatant at 390 nm, with the content of H_2_O_2_ expressed as μmol g^–1^.

### Measurement of enzymatic and non-enzymatic antioxidants and related gene expression

The specific enzyme activity detection kit purchased by Nanjing Jiancheng Bioengineering Institute facilitated to calculate the activities of SOD, CAT, and POD, using 0.1 g of samples individually. The activity (1 U) of SOD was defined as the number of enzymes required to reduce nitrotetrazolium blue chloride (NBT) to half of that of the control group. The activity (1 U) of CAT was defined as a 0.1 absorbance reduction at 240 nm, POD activity (1 U) defined as a 0.01 absorbance reduction at 470 nm. While the SOD, CAT, and POD activities were expressed as U g^−1^. The application of The method of Li determined The contents of ASA and GSH. The contents of ASA and GSH were μmol g^−1^.^31^ The relative expression levels of *sod1, cat1*, and *cevi16* genes were completely determined by Shanghai Sheng Gong Biological Engineering Co., Ltd with separate 10 g samples.

#### Statistical analysis

The experimental data were statistically analyzed using one-factor analysis of variance (*ANOVA*). Analysis of the significance of differences between control and each treatment was performed by the *Duncan multiple-range test* at the 0.05 level of confidence using SPSS statistical software (SPSS, Chicago, IL, USA). The data of each index in the figure were presented as the mean ± standard deviation (SD) of three biological replications, with three samples of each replication. Calculations were performed using *Microsoft Excel* (*Microsoft*, USA). Graphs were plotted using GraphPad Prism 7.05 (GraphPad Software, San Diego, CA, USA).

## Results

### Photosynthetic pigments

**Figure 1.**
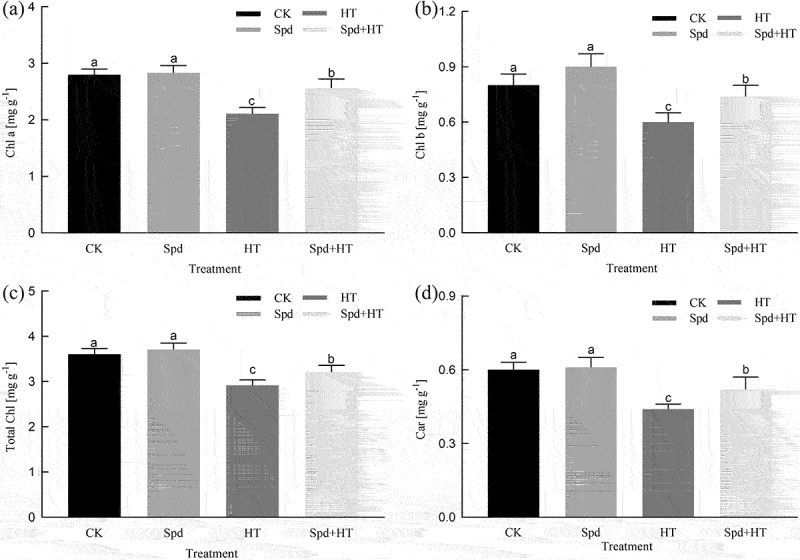
Effects of exogenous spermidine treatment on chlorophyll and carotenoid concentrations of citrus plants exposed to high temperature stress. Note: Different letters indicate statistically significant by Duncan multiple- range test at P < .05. Data were expressed as the mean ± standard error of three independent biological replicates. Key: Chl – chlorophyll, Car – carotenoid.

High-temperature stress caused a sharp decrease in the pigment contents (Figure 1). The Chl*a*, Chl*b*, total Chl and Car contents in only the heat-stressed seedlings decreased by 25%, 25%, 19%, and 27%, respectively, in contrast with their corresponding controls. Conversely, the application of exogenous melatonin to heat-stressed seedlings led to the restoration of the Chl*a*, Chl*b*, total Chl, and Car contents compared to citrus seedlings subjected to HT stress, which amounted to 21%, 23%, 9%, and 18%, respectively.

### Light response curves

**Figure 2.**
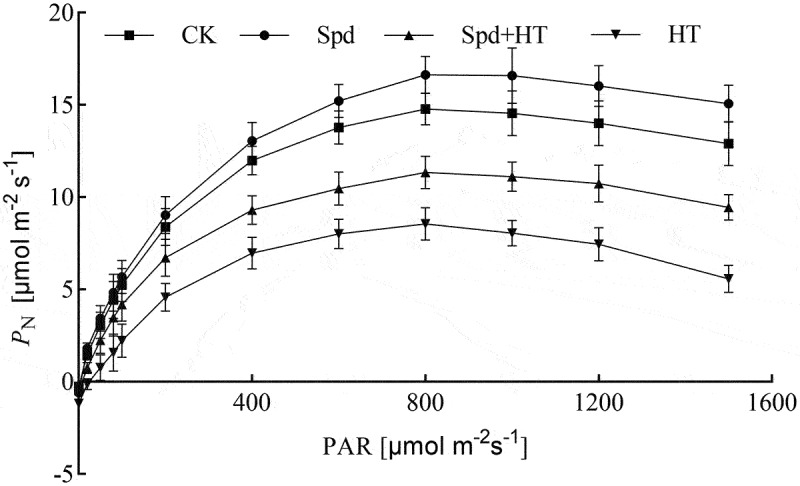
Effects of exogenous spermidine treatment on light response curves of citrus plants exposed to high temperature stress. Note: Data were expressed as the mean ± standard error of three independent biological replicates. Key: PN – net photosynthetic rate, PAR – photosynthetically active radiation.

Light response curves (LRCs) ranging from 0 to 1500 µmol m^–2^ s^–1^ were used to investigate the photosynthetic capacity of the citrus plants (Figure 2). With the increase in light intensity, LRCs in citrus leaves increased rapidly. However, the LRCs became steady after the light intensity reached 800 µmol m^–2^ s^–1^. The CK and Spd group showed the higher LRC, while LRC under HT was only 43% of the control when the light intensity reached 1500 µmol m^–2^ s^–1^. Application of Spd effectively improved the LRC under HT treatment, as exhibited by the middle curve.

As shown in Table 1. HT stress led to a decline in *P*_Nmax_, AQE, and LSP, but an increase in LCP. Compared with the control, the *P*_Nmax_, AQE, and LSP in citrus leaves under HT treatment decreased by 64%, 44%, and 32%, respectively, and the LCP increased by 40%. However, exogenous application of Spd significantly increased *P*_Nmax_, AQE, and LSP under HT conditions in citrus by 44%, 54%, and 23%, respectively, but reduced LCP by 9%.

**Table 1.** Effects of exogenous spermidine on photosynthetic characteristic parameters in citrus seedling leaves under high-temperature stress

Treatment	*P*_Nmax_ [μmol m^–2^ s^–1^]	AQE	LCP [μmol m^–2^ s^–1^]	LSP [μmol m^–2^ s^–1^]
CK	15.86 ± 0.22^a^	0.039 ± 0.002^a^	8.29 ± 0.29^a^	906.75 ± 19.65^a^
Spd	15.92 ± 0.16^a^	0.040 ± 0.003^a^	8.33 ± 0.33^a^	914.64 ± 13.67^a^
HT	5.79 ± 0.20^c^	0.022 ± 0.001^c^	11.58 ± 0.23^c^	621.36 ± 24.99^c^
Spd + HT	8.33 ± 0.21^b^	0.034 ± 0.002^b^	9.33 ± 0.14^b^	764.44 ± 20.23^b^

Note: For each variable, *different lowercase letters* indicate statistically significant by *Duncan multiple- range test* at P < 0.05. Data were expressed as the mean ± standard error of three independent biological replicates. Key: *P*_Nmax_ – maximum photosynthetic rate, AQE – apparent quantum efficiency, LCP – light compensation point, LSP – light saturation point.

### Gas exchange parameters

**Figure 3.**
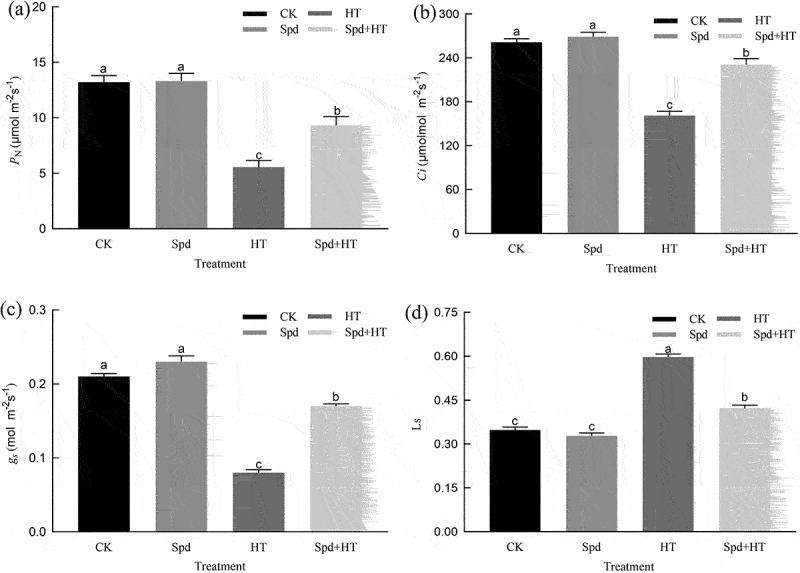
Effects of exogenous spermidine treatment on gas exchange parameters of citrus plants exposed to high temperature stress. Note: Different letters indicate statistically significant by Duncan multiple- range test at P < .05. Data were expressed as the mean ± standard error of three independent biological replicates. Key: PN – net photosynthetic rate, gs – stomatal conductance, Ci – intercellular carbon dioxide concentration, Ls – stomatal limitation value.

The values of *P*_N_, *C*_i,_ and *g*_s_ significantly decreased in both heat-stressed citrus seedlings with and those without Spd treatment compared to non-stressed plants (Figure 3). However, the reductions were more conspicuous in the seedlings exposed only to heat stress. The Spd-treated seedlings reversed the inhibitory effects of high temperatures on those parameters, with *P*_N_, *C*_i,_ and *g*_s_ levels substantially increased by 68%, 43%, and 95%, respectively, compared with untreated stressed seedlings. Conversely, the Ls in the Spd-treated plants under heat stress sharply elevated by 29%, compared with those of the plants subjected to heat stress alone. In addition, no statistically significant differences observed between the control and Spd-only seedlings regarding these gas exchange parameters.

### Chl fluorescence parameters

**Figure 4.**
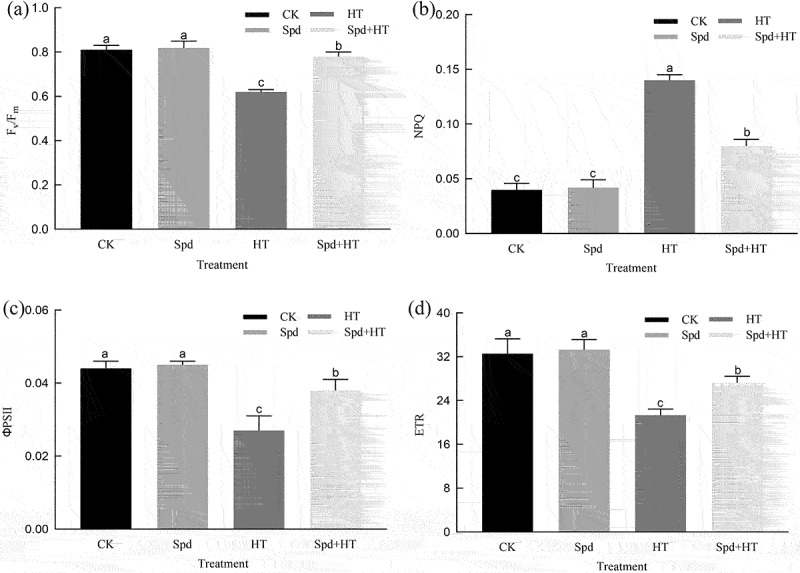
Effects of exogenous spermidine treatment on Chl fluorescence parameters of citrus plants exposed to high temperature stress. Note: Different letters indicate statistically significant by Duncan multiple- range test at P < .05. Data were expressed as the mean ± standard error of three independent biological replicates. Key: Fv/Fm – maximal quantum yield of PSII photochemistry, NPQ – nonphotochemical quenching, ΦPSII – effective quantum yield of PSII photochemistry, ETR – electron transport rate.

As shown in Figure 4, under normal growth conditions, Spd had no significant effects on the F_v_/F_m_, ΦPSII, NPQ, and ETR values. However, heat stress significantly inhibited the F_v_/F_m_, ΦPSII, and ETR values by 23%, 250%, and 61%, respectively, and increased the NPQ value 3 times. In addition, the values of NPQ decreased while the values of F_v_/F_m_, ΦPSII, and ETR increased with Spd supplementation in citrus seedlings exposed to heat stress.

### H_2_O_2_ contents and lipid peroxidation

As shown in Table 2, under normal growth conditions, the addition of Spd had no significant effects on H_2_O_2_ and MDA contents. HT stress progressively increased the contents of H_2_O_2_ and MDA in citrus leaves. Compared to CK, H_2_O_2_ and MDA contents increased significantly by 2 times and 2.44 times, respectively. On the contrary, spraying exogenous Spd can effectively alleviate the damage to citrus seedlings under high-temperature stress. Spd-treated plants under heat stress resulted in a significant decrease in H_2_O_2_ and MDA contents (52.4% and 72.4%, respectively) compared to stress treatment with HT alone.

**Table 2.** Effects of exogenous spermidine (Spd) on MDA and H_2_O_2_ contents in citrus seedling leaves under high-temperature stress

Treatment	MDA content	H_2_O_2_ content
	[nmol g^−1^]	[μmol g^−1^]
CK	28.74 ± 2.13^c^	51.89 ± 4.25^c^
Spd	28.38 ± 2.22^c^	52.33 ± 2.10^c^
HT	98.03 ± 3.71^a^	102.32 ± 6.44^a^
Spd + HT	42.05 ± 2.34^b^	67.66 ± 3.19^b^

Note: For each variable, *different lowercase letters* indicate statistically significant by *Duncan multiple- range test* at P < 0.05. Data were expressed as the mean ± standard error of three independent biological replicates. Key: MDA – malondialdehyde, H_2_O_2_ – hydrogen peroxide.

### Antioxidant enzyme activities and related gene expression

**Figure 5.**
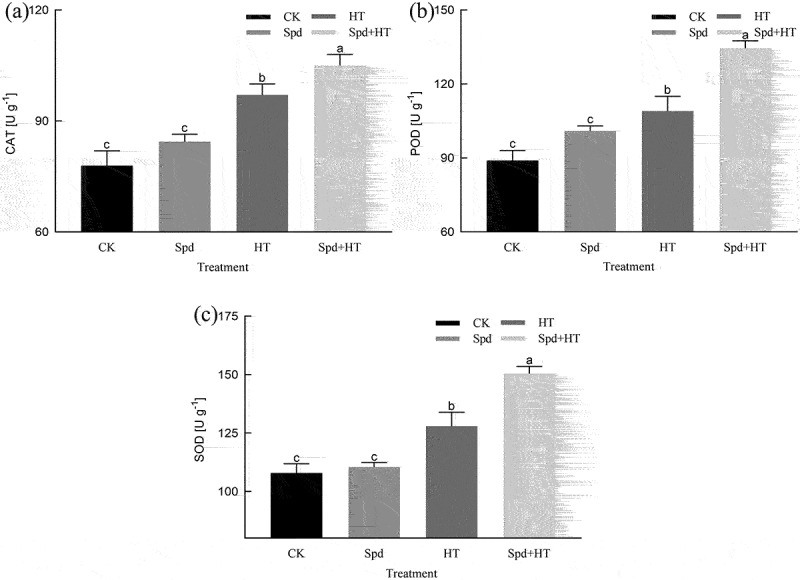
Effects of exogenous spermidine treatment on the activity of SOD, POD and CAT in citrus plants exposed to high temperature stress. Note: Different letters indicate statistically significant by Duncan multiple- range test at P < .05. Data were expressed as the mean ± standard error of three independent biological replicates. Key: SOD – superoxide dismutase, POD – peroxidase, CAT – catalase.

HT stress led to significant regulation of antioxidant defense in citrus leaves. Activities of antioxidant enzymes (SOD, POD, and CAT) increased in citrus plants due to the imposition of HT than those in non-stressed plants (Figure 5). However, exogenously applied Spd caused a further increase in antioxidant enzyme activity of stressed plants. Furthermore, spraying the seedlings with exogenous Spd enhanced the activities of antioxidant enzymes in non-stressed plants as well. In addition, with the application of exogenous Spd under HT stress, the gene expression of *sod1, cevi16, and cat1* was significantly promoted by 19.6%, 27.6%, and 48.19% compared with the control group, respectively (Table 3) .

**Table 3.** Effects of exogenous spermidine (Spd) on the transcript level of *sod1, cevi16*, and *cat1* in citrus seedling leaves under high-temperature stress

Treatment	*sod1* relative expression	*cevi16* relative expression	*cat1* relative expression
CK	5.34 ± 0.03^d^	1.46 ± 0.02^d^	1.84 ± 0.02^d^
Spd	5.88 ± 0.02 ^c^	1.73 ± 0.02^c^	2.03 ± 0.01^c^
HT	6.73 ± 0.04^b^	2.32 ± 0.01^b^	3.32 ± 0.04^b^
Spd + HT	8.05 ± 0.03^a^	2.96 ± 0.03^a^	4.92 ± 0.01^a^

Note: For each variable, *different lowercase letters* indicate statistically significant by *Duncan multiple- range test* at P < 0.05. Data were expressed as the mean ± standard error of three independent biological replicates. Key: *sod1* – superoxide dismutase 1 gene, *cevi16* – peroxidase gene, *cat1* – catalase 1 gene.

### Ascorbic acid and glutathione contents

As shown in Figure 5, spraying Spd under normal temperature increased the ASA content, albeit with little change, and there was no significant difference in ASA content compared with that in the control treatment. Under HT stress conditions, the ASA content tended to increase, which was 0.75 times that of the normal temperature controls. Exogenously applied Spd under HT stress increased the ASA content and there was no significant difference in ASA content compared with that in the HT treatment. The variation trend of GSH content under HT stress and in response to Spd spraying was consistent with the variation in ASA content.

## Discussion

Chlorophyll is the basic unit of plant energy systems during the process of photosynthesis.^32^ However, Chl is very fragile and easily destroyed by HT stress, which has been confirmed by many other authors,^33–35^ and also has been presented in our experiments (Figure 1), probably due to, under HT stress, the structure and function of Chl were severely destroyed, and the activity of Chl degrading enzyme was significantly increased. However, the addition of exogenous Spd could enhance photosynthetic pigment-synthesis or slow its degradation rate.^36^ The results were consistent with these reports. Photosynthesis is highly sensitive to high-temperature stress and is often inhibited before other cell functions are impaired.^37,38^ Previous research demonstrated that the stomatal limiting factors causes a decrease in photosynthetic rate.^39^ The suggested mechanism is due to the reduction of leaf *g*_s_, by hindering the supply of CO_2_ in chloroplasts.^40^ However, some studies believed that the inhibition of photosynthesis was caused by non-stomatal factors, which was reflected by the higher CO_2_ concentration in mesophyll cells, but the lower affinity of Rubisco for CO_2_.^41^ In our study, *P*_N_ decreased, accompanied by different degrees of *g*_s_ and *C*_i_ increased and L_s_ decreased significantly under heat stress (Figure 3), which indicated that the decrease of *P*_N_ might be due to the effect of non-stomatal factors. However, with the application of Spd, the *P*_N_ increased and the values of L_s_ reduced under HT conditions. The underlying mechanism might be due to Spd treatment promoting the transport of CO_2_, the protein and gene expression of Ribulose-1,5-Bisphosphate Carboxylase (Rubisco) and phenol pyruvate carboxylase.^42^ In addition, HT also significantly influenced the photosynthetic curve characteristic parameters, such as reduced *P*_Nmax_, AQE, and LCP, and significantly increasing the LSP (Table 1), which was consistent with the research results by Zhou *et al*. (2015),^43^ but the application of Spd could effectively alleviate the influence of HT stress on the characteristic parameters of the light response curve.

Chlorophyll fluorescence technique is a practical method to study the plant photosynthetic apparatus, especially the function of PSII.^44,45^ Chl fluorescence signal and its measured parameters have been successfully used to probe and elucidate injury to the PSII from various stresses.^38^ F_v_/F_m_ represents the maximal quantum yield of PSII photochemistry, and its value is very stable, between 0.78 and 0.84. In our study, the F_v_/F_m_ decreased in the HT-stressed leaves, which implied the quantum of absorption into the heart and fluorescence increased, and the absorption quantum converted into chemical fixation energy decreased. The treatment with exogenous Spd before the HT stress, significantly alleviated the decrease of F_v_/F_m_ and ETR, which indicated that Spd might alleviate the effect of HT on the photosynthetic process by improving the photosynthetic electron transport.^46^ Moreover, the ΦPSII decreased, whereas NPQ increased under HT stress. This indicates that the electron transfer of photosynthetic electrons was blocked, and the efficiency of photosynthetic pigments converting light energy into chemical energy decreased.^47,48^ Pretreatment with exogenous Spd under HT stress could improve ΦPSII, but reduce NPQ, which might be that exogenous Spd could protect PSII from excessive energy damage, and enhance the stability of the reaction center of the photosystem,^18^ which is conducive to the conversion of light energy into chemical energy, thereby providing enough energy for carbon assimilation.

The effect of HT stress on chlorophyll and photosynthetic apparatus suggested to relating to the production of ROS^49,50^ Under abiotic stress conditions, the production of ROS can exceed the scavenging capability of the cell, which has the potential of damaging the biomolecules leading to cell death.^51^ MDA is one of the most known secondary products of lipid peroxidation, and it can be used as a marker of cell membrane injury.^52^ Exogenous Spd treatment effectively inhibits the accumulation of H_2_O_2_ and MDA in plants under HT stress, which has been confirmed in wheat, rice, and other plants.^53,54^ In this study, we observed that H_2_O_2_ and MDA contents significantly increased under HT stress, suggesting cell membrane damage; however, H_2_O_2_ and MDA contents decreased significantly when exogenous Spd was spayed under HT stress. A reduction of H_2_O_2_ and MDA suggested that exogenous Spd showed a protective effect against membrane damage under HT stress.

To reduce oxidative stress, plants have developed a series of both enzymatic and non-enzymatic detoxification systems to deal with ROS, controlling the formation and removal rates, and thereby protecting cells from oxidative damage.^55,56^ The antioxidant enzyme system, comprising SOD, POD, and CAT, is an important system that protects cells from the negative effects of ROS.^57^ Our experimental results showed that under HT treatment, SOD, POD, and CAT activities in leaves showed varying degrees of increase (Figure 4). These results suggested that to protect themselves from HT stress, plants increase the activity of their antioxidant enzymes, thereby reducing adversity stress, which is consistent with previous research results.^58,59^ Our research confirmed that Spd was involved in ROS scavenging under HT conditions. Therefore, the reversal of HT stress by Spd is due to its ability in improving antioxidant capacity and increasing the scavenging of ROS.

**Figure 6.**
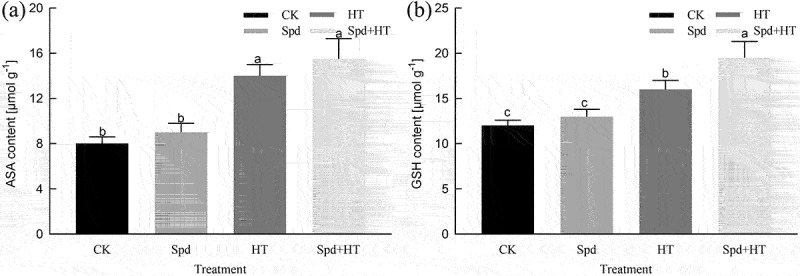
Effects of exogenous spermidine treatment on the content of ASA and GSH in citrus plants exposed to high temperature stress. Note: Different letters indicate statistically significant by Duncan multiple- range test at P < .05. Data were expressed as the mean ± standard error of three independent biological replicates. Key: ASA – Ascorbic acid, GSH – glutathione, CAT – catalase.

The AsA-GSH cycle is an important non-enzymatic system in plants that maintains the oxidative environment in cells by regulating the content of both AsA and GSH and plays a protective role in plants.^60^ The AsA is an effective active oxygen scavenger^61^ and GSH is widely distributed in plant tissues. It can participate directly or indirectly in the detoxification of ROS.^62^ The GSH is meanwhile involved in methylglyoxal (MG) detoxification.^62^ In this study, the AsA and GSH contents in citrus leaves tended to increase under HT stress (Figure 6), which was consistent with the results of the study on the effect of heat stress on lettuce,^19^ and the levels of these two antioxidants further increased after the application of exogenous Spd. Our results showed that exogenous Spd could increase the heat resistance of citrus seedlings under HT stress by increasing AsA and GSH levels.

## Conclusion

HT stress negatively influences photosynthesis and the chlorophyll fluorescence parameters in citrus seedlings. The application of exogenous Spd improved photosynthesis, maintained the balance in ROS metabolism and alleviated the damage caused by HT stress. In addition, the results also indicated that exogenous Spd could alleviate the damage of citrus seedlings under HT stress by improving photosynthetic efficiency, enhancing the activities of enzymatic and non-enzymatic antioxidants, and reducing oxidative stress in leaves. Therefore, exogenous Spd treatment might be a suitable approach to improve the tolerance of citrus seedlings under HT stress.

## References

[1] Raza A, Razzaq A, Mehmood SS, Zou X, Zhang X, Lv Y, Xu J. Impact of climate change on crops adaptation and strategies to tackle its outcome: a review. Plants. 2019;8:34. doi:10.3390/plants8020034. PMC6409995 30704089

[2] Mittler R, Finka A, Goloubinoff P. How do plants feel the heat? Trends Biochem Sci. 2012;37:118–10. doi:10.1016/j.tibs.2011.11.007. 22236506

[3] Zhang JH, HUANG WD, LIU YP, PAN QH. Effects of temperature acclimation pretreatment on the ultrastructure of mesophyll cells in young grape plants (Vitis vinifera L. cv. Jingxiu) under cross‐temperature stresses. J Integr Plant Biol. 2005;47:959–970. doi:10.1111/j.1744-7909.2005.00109.x.

[4] Song Y, Chen Q, Ci D, Shao X, Zhang D. Effects of high temperature on photosynthesis and related gene expression in poplar. Bmc Plant Biol. 2014;14:1–20. doi:10.1186/1471-2229-14-111. 24774695 PMC4036403

[5] Gerganova MT, Faik AK, Velitchkova MY. Acquired tolerance of the photosynthetic apparatus to photoinhibition as a result of growing Solanum lycopersicum at moderately higher temperature and light intensity. Funct Plant Biol. 2019;46:555–566. doi:10.1071/FP18264. 30940333

[6] Muhlemann JK, Younts TL, Muday GK. Flavonols control pollen tube growth and integrity by regulating ROS homeostasis during high-temperature stress. PNAS. 2018;115:E11188–E11197. doi:10.1073/pnas.1811492115. 30413622 PMC6255205

[7] Chung IM, Kim JJ, Lim JD, Yu CY, Kim SH, Hahn SJ. Comparison of resveratrol, SOD activity, phenolic compounds and free amino acids in rehmannia glutinosa under temperature and water stress. Environ Exp Bot. 2006;56:44–53. doi:10.1016/j.envexpbot.2005.01.001.

[8] Xu C, Yang ZQ, Yang SQ, Wang L, Wang MT. High humidity alleviates photosynthetic inhibition and oxidative damage of tomato seedlings under heat stress. Photosynthetica. 2020;58:146–155. doi:10.32615/ps.2019.168.

[9] Li L, Gu W, Li C, Li W, Li C, Li J, Wei S. Exogenous spermidine improves drought tolerance in maize by enhancing the antioxidant defence system and regulating endogenous polyamine metabolism. Crop Pasture Sci. 2018;69:1076–1091. doi:10.1071/CP18271.

[10] Mir MA, John R, Alyemeni MN, Alam P, Ahmad P. Jasmonic acid ameliorates alkaline stress by improving growth performance, ascorbate glutathione cycle and glyoxylase system in maize seedlings. Sci Rep. 2018;8:2831. doi:10.1038/s41598-018-21097-3. 29434207 PMC5809373

[11] Hussain SS, Ali M, Ahmad M, Siddique KH. Polyamines: natural and engineered abiotic and biotic stress tolerance in plants. Biotechnol Adv. 2011;29:300–311. doi:10.1016/j.biotechadv.2011.01.003. 21241790

[12] Kusano T, Yamaguchi K, Berberich T, Takahashi Y. Advances in polyamine research in 2007. J Plant Res. 2007;120:345–350. doi:10.1007/s10265-007-0074-3. 17351711

[13] Shi H, Chan Z. Improvement of plant abiotic stress tolerance through modulation of the polyamine pathway. J Integr Plant Biol. 2014;56:114–121. doi:10.1111/jipb.12128. 24401132

[14] Galston AW, Kaur Sawhney R, Altabella T, Tiburcio AF. Plant polyamines in reproductive activity and response to abiotic stress. Bot Acta. 1997;110:197–207. doi:10.1111/j.1438-8677.1997.tb00629.x.

[15] Tiburcio AF, Alcazar R. Potential applications of polyamines in agriculture and plant biotechnology. Polyamines. 2018;40:489–508. 10.1007/978-1-4939-7398-9_40 29080190

[16] Killiny N, Nehela Y. Citrus polyamines: structure, biosynthesis, and physiological functions. Plants. 2020;9:426. doi:10.3390/plants9040426. PMC7238152 32244406

[17] Pottosin I, Velarde-Buendía AM, Bose J, Fuglsang AT, Shabala S. Polyamines cause plasma membrane depolarization, activate Ca2+-, and modulate H+-ATPase pump activity in pea roots. J Exp Bot. 2014;65:2463–2472. doi:10.1093/jxb/eru133. 24723394

[18] Jing JG, Guo SY, Li YF, Li WH. Effects of polyamines on agronomic traits and photosynthetic physiology of wheat under high temperature stress. Photosynthetica. 2019;57:912–920. doi:10.32615/ps.2019.104.

[19] Li C, Han Y, Hao J, Qin X, Liu C, Fan S. Effects of exogenous spermidine on antioxidants and glyoxalase system of lettuce seedlings under high temperature. Plant Signal Behav. 2020;15:1824697. doi:10.1080/15592324.2020.1824697. 32985921 PMC7671048

[20] Yang X, Han Y, Hao J, Qin X, Liu C, Fan S. Exogenous spermidine enhances the photosynthesis and ultrastructure of lettuce seedlings under high-temperature stress. Sci Hortic-Amsterdam. 2022;291:110570. doi:10.1016/j.scienta.2021.110570.

[21] Zhou H, Guo S, An Y, Shan X, Wang Y, Shu S, Sun J. Exogenous spermidine delays chlorophyll metabolism in cucumber leaves (Cucumis sativus L.) under high temperature stress. Acta Physiol Plant. 2016;38:1–12. doi:10.1007/s11738-016-2243-2.

[22] Zandalinas SI, Balfagón D, Arbona V, Gómez-Cadenas A. Modulation of antioxidant defense system is associated with combined drought and heat stress tolerance in citrus. Front Plant Sci. 2017;8:953. doi:10.3389/fpls.2017.00953. 28638395 PMC5461256

[23] Balfagón D, Zandalinas SI, Gómez Cadenas A. High temperatures change the perspective: integrating hormonal responses in citrus plants under co‐occurring abiotic stress conditions. Physiol Plant. 2019;165:183–197. doi:10.1111/ppl.12815. 30091288

[24] Arnon DI. Copper enzymes in isolated chloroplasts. polyphenoloxidase in beta vulgaris. Plant Physiol. 1949;24:1. doi:10.1104/pp.24.1.1. 16654194 PMC437905

[25] Yang ZQ, Xu C, Wang MT, Zhao HL, Zheng YJ, Huang HJ, Vuguziga F, Umutoni MA. Enhancing the thermotolerance of tomato seedlings by heat shock treatment. Photosynthetica. 2019;57:1184–1192. doi:10.32615/ps.2019.127.

[26] Herrmann HA, Schwartz J, Johnson GN. From empirical to theoretical models of light response curves-linking photosynthetic and metabolic acclimation. Photosynth Res. 2020;145:5–14. doi:10.1007/s11120-019-00681-2. 31654195 PMC7308256

[27] Farquhar J, Savarino J, Airieau S, Thiemens MH. Observation of wavelength‐sensitive mass‐independent sulfur isotope effects during SO2 photolysis: implications for the early atmosphere. J Geophys Res: Planets. 2001;106:32829–32839. doi:10.1029/2000JE001437.

[28] Jan S, Alyemeni MN, Wijaya L, Alam P, Siddique KH, Ahmad P. Interactive effect of 24-epibrassinolide and silicon alleviates cadmium stress via the modulation of antioxidant defense and glyoxalase systems and macronutrient content in Pisum sativum L. seedlings. Bmc Plant Biol. 2018;18:1–18. doi:10.1186/s12870-018-1359-5. 30012086 PMC6048797

[29] Hodges DM, DeLong JM, Forney CF, Prange RK. Improving the thiobarbituric acid-reactive-substances assay for estimating lipid peroxidation in plant tissues containing anthocyanin and other interfering compounds. Planta. 1999;207:604–611. doi:10.1007/s004250050524. 28456836

[30] Wang DY, Wang J, Shi SH, Huang LX, Zhu M, Li FH. Exogenous melatonin ameliorates salinity-induced oxidative stress and improves photosynthetic capacity in sweet corn seedlings. Photosynthetica. 2021;59:327–336. doi:10.32615/ps.2021.031.

[31] Li Y, Liu Y, Zhang J. Advances in the research on the AsA-GSH cycle in horticultural crops. Front Agric China. 2010;4(1):84–90. doi:10.1007/s11703-009-0089-8.

[32] Palta JP. Leaf chlorophyll content. Remote Sens Rev. 1990;5:207–213. doi:10.1080/02757259009532129.

[33] Tian J, Wang L, Yang Y, Sun J, Guo S. Exogenous spermidine alleviates the oxidative damage in cucumber seedlings subjected to high temperatures. J Am Soc Hortic Sci. 2012;137:11–19. doi:10.21273/JASHS.137.1.11.

[34] Mostofa MG, Yoshida N, Fujita M. Spermidine pretreatment enhances heat tolerance in rice seedlings through modulating antioxidative and glyoxalase systems. Plant Growth Regul. 2014;73:31–44. doi:10.1007/s10725-013-9865-9.

[35] Zhang L, Hu T, Amombo E, Wang G, Xie Y, Fu J. The alleviation of heat damage to photosystem II and enzymatic antioxidants by exogenous spermidine in tall fescue. Front Plant Sci. 2017;8:1747. doi:10.3389/fpls.2017.01747. 29075277 PMC5644155

[36] Khoshbakht D, Asghari MR, Haghighi M. Effects of foliar applications of nitric oxide and spermidine on chlorophyll fluorescence, photosynthesis and antioxidant enzyme activities of citrus seedlings under salinity stress. Photosynthetica. 2018;56:1313–1325. doi:10.1007/s11099-018-0839-z.

[37] Berry J, Bjorkman O. Photosynthetic response and adaptation to temperature in higher plants. Annu Rev Physiol. 1980;31:491–543. doi:10.1146/annurev.pp.31.060180.002423.

[38] Mathur S, Agrawal D, Jajoo A. Photosynthesis: response to high temperature stress. J Photochem Photobiol B: Biol. 2014;137:116–126. doi:10.1016/j.jphotobiol.2014.01.010. 24796250

[39] Farquhar GD, Sharkey TD. Stomatal conductance and photosynthesis. Annu Rev Physiol. 1982;33:317–345. doi:10.1146/annurev.pp.33.060182.001533.

[40] Muraoka H, Tang Y, Terashima I, Koizumi H, Washitani I. Contributions of diffusional limitation, photoinhibition and photorespiration to midday depression of photosynthesis in Arisaema heterophyllum in natural high light. Plant Cell Environ. 2000;23:235–250. doi:10.1046/j.1365-3040.2000.00547.x.

[41] Pilon C, Snider JL, Sobolev V, Chastain DR, Sorensen RB, Meeks CD, Massa AN, Walk T, Singh B, Earl HJ. Assessing stomatal and non-stomatal limitations to carbon assimilation under progressive drought in peanut (Arachis hypogaea L.). J Plant Physiol. 2018;231:124–134. doi:10.1016/j.jplph.2018.09.007. 30261481

[42] Naeem M, Traub JR, Athar H, Loescher W. Exogenous calcium mitigates heat stress effects in common bean: a coordinated impact of photoprotection of PSII, up-regulating antioxidants, and carbohydrate metabolism. Acta Physiol Plant. 2020;42:1–13. doi:10.1007/s11738-020-03171-4.

[43] Zhou R, Yu X, Kjær KH, Rosenqvist E, Ottosen C, Wu Z. Screening and validation of tomato genotypes under heat stress using Fv/Fm to reveal the physiological mechanism of heat tolerance. Environ Exp Bot. 2015;118:1–11. doi:10.1016/j.envexpbot.2015.05.006.

[44] Kalaji HM, Schansker G, Brestic M, Bussotti F, Calatayud A, Ferroni L, Goltsev V, Guidi L, Jajoo A, Li P. Erratum to: frequently asked questions about chlorophyll fluorescence, the sequel. Photosynth Res. 2017;132:67. doi:10.1007/s11120-017-0356-0. 27815801 PMC5357263

[45] Maxwell K, Johnson GN. Chlorophyll fluorescence—a practical guide. J Exp Bot. 2000;51:659–668. doi:10.1093/jexbot/51.345.659. 10938857

[46] Poudyal D, Rosenqvist E, Ottosen C. Phenotyping from lab to field–tomato lines screened for heat stress using Fv/Fm maintain high fruit yield during thermal stress in the field. Funct Plant Biol. 2018;46:44–55. doi:10.1071/FP17317. 30939257

[47] Jahan MS, Guo S, Sun J, Shu S, Wang Y, Abou El-Yazied A, Alabdallah NM, Hikal M, Mohamed MH, Ibrahim MF. Melatonin-mediated photosynthetic performance of tomato seedlings under high-temperature stress. Plant Physiol Bioch. 2021;167:309–320. doi:10.1016/j.plaphy.2021.08.002. 34392044

[48] Yuan L, Yuan Y, Liu S, Wang J, Zhu S, Chen G, Hou J, Wang C. Influence of high temperature on photosynthesis, antioxidative capacity of chloroplast, and carbon assimilation among heat-tolerant and heat-susceptible genotypes of nonheading Chinese cabbage. Hortscience. 2017;52:1464–1470. doi:10.21273/HORTSCI12259-17.

[49] Camejo D, Jiménez A, Alarcón JJ, Torres W, Gómez JM, Sevilla F. Changes in photosynthetic parameters and antioxidant activities following heat-shock treatment in tomato plants. Funct Plant Biol. 2006;33:177–187. doi:10.1071/FP05067. 32689224

[50] Guo Y, Zhou H, Zhang L. Photosynthetic characteristics and protective mechanisms against photooxidation during high temperature stress in two citrus species. Sci Hortic-Amsterdam. 2006;108:260–267. doi:10.1016/j.scienta.2006.01.029.

[51] Gill SS, Tuteja N. Reactive oxygen species and antioxidant machinery in abiotic stress tolerance in crop plants. Plant Physiol Bioch. 2010;48:909–930. 10.1016/j.plaphy.2010.08.016 20870416

[52] Rahman M, Rahman K, Sathi KS, Alam M, Nahar K, Fujita M, Hasanuzzaman M. Supplemental selenium and boron mitigate salt-induced oxidative damages in glycine max L. Plants. 2021;10:2224. doi:10.3390/plants10102224. 34686033 PMC8539870

[53] Jing J, Guo S, Li Y, Li W. The alleviating effect of exogenous polyamines on heat stress susceptibility of different heat resistant wheat (Triticum aestivum L.) varieties. Sci Rep-Uk. 2020;10:1–12. 10.1038/s41598-020-64468-5 PMC7198572 32366860

[54] Tang S, Zhang H, Li L, Liu X, Chen L, Chen W, Ding Y. Exogenous spermidine enhances the photosynthetic and antioxidant capacity of rice under heat stress during early grain-filling period. Funct Plant Biol. 2018;45:911–921. doi:10.1071/FP17149. 32291055

[55] Foyer CH, Noctor G. Redox sensing and signalling associated with reactive oxygen in chloroplasts, peroxisomes and mitochondria. Physiol Plant. 2003;119:355–364. doi:10.1034/j.1399-3054.2003.00223.x.

[56] Mittler R. Oxidative stress, antioxidants and stress tolerance. Trends Plant Sci. 2002;7:405–410. doi:10.1016/S1360-1385(02)02312-9. 12234732

[57] Noctor G, Foyer CH. Ascorbate and glutathione: keeping active oxygen under control. Annu Rev Plant Biol. 1998;49:249–279. doi:10.1146/annurev.arplant.49.1.249. 15012235

[58] Almeselmani M, Deshmukh PS, Sairam RK, Kushwaha SR, Singh TP. Protective role of antioxidant enzymes under high temperature stress. Plant Sci. 2006;171:382–388. doi:10.1016/j.plantsci.2006.04.009. 22980208

[59] Zhang X, Ervin EH, Schmidt RE. Plant growth regulators can enhance the recovery of Kentucky bluegrass sod from heat injury. Crop Sci. 2003;43:952–956. doi:10.2135/cropsci2003.9520.

[60] Bartoli CG, Buet A, Gergoff Grozeff G, Galatro A, Simontacchi M. Ascorbate-glutathione cycle and abiotic stress tolerance in plants.In. In: Hossain M, Munné-Bosch S, Burritt D, Diaz-Vivancos P, Fujita M, Lorence A, editors. Ascorbic acid in plant growth, development and stress tolerance. Cham: Springer International Publishing; 2017. p. 177–200.

[61] Foyer CH, Noctor G. Ascorbate and glutathione: the heart of the redox hub. Plant Physiol. 2011;155:2–18. doi:10.1104/pp.110.167569. 21205630 PMC3075780

[62] Hasanuzzaman M, Nahar K, Hossain M, Mahmud J, Rahman A, Inafuku M, Oku H, Fujita M. Coordinated actions of glyoxalase and antioxidant defense systems in conferring abiotic stress tolerance in plants. Int J Mol Sci. 2017;18:200. doi:10.3390/ijms18010200. PMC5297830 28117669

